# *In-silico* prediction of highly promising natural fungicides against the destructive blast fungus *Magnaportheoryzae*

**DOI:** 10.1016/j.heliyon.2023.e15113

**Published:** 2023-04-10

**Authors:** Md Abdullah Al Mamun Khan, Asif Ahsan, Md Arif Khan, Jannatul Maowa Sanjana, Suvro Biswas, Md Abu Saleh, Dipali Rani Gupta, M. Nazmul Hoque, Tahsin Islam Sakif, Md Masuder Rahman, Tofazzal Islam

**Affiliations:** a Department of Biotechnology and Genetic Engineering, Mawlana Bhashani Science and Technology University, Tangail 1902, Bangladesh; b Department of Biotechnology, Bangladesh Agricultural University, Mymensingh, 2202, Bangladesh; c Department of Biotechnology and Genetic Engineering, University of Development Alternative, Dhaka 1209, Bangladesh; d Microbiology Laboratory, Department of Genetic Engineering and Biotechnology, University of Rajshahi, Rajshahi 6205, Bangladesh; e Institute of Biotechnology and Genetic Engineering (IBGE), Bangabandhu Sheikh Mujibur Rahman Agricultural University (BSMRAU), Gazipur 1706, Bangladesh; f Department of Gynecology, Obstetrics and Reproductive Health, BSMRAU, Gazipur 1706, Bangladesh; g Lane Department of Computer Science and Electrical Engineering, West Virginia University, Morgantown, WV 26506-6109, USA; h Bio-Bio-1 Bioinformatics Research Foundation, Dhaka, Bangladesh

**Keywords:** Molecular docking, Antifungal metabolites, ssDNA, Protein, *Magnaporthe oryzae*

## Abstract

*Magnaporthe oryzae* causes destructive blast disease in more than 50 species of the major cereal crops rice, wheat and maize and destroys food of millions of people worldwide. Application of synthetic chemical fungicides are environmentally hazardous and unreliable in controlling *M. oryzae*. Conversely, naturally occurring biofungicides with multiple modes of actions are needed to be discovered for combatting the blast fungus. To find the effective biofungicides, we performed molecular docking study of some potential antifungal natural compounds targeting two proteins including a single-stranded DNA binding protein *MoSub1* (4AGH), and an effector protein AVR-Pik (5E9G) of *M. oryzae* that regulates transcription in fungus and/or suppresses the host cell immunity. The thirty-nine natural compounds previously shown to inhibit *M. oryzae* growth and reproduction were put under molecular docking against these two proteins followed by simulation, free energy, and interaction analysis of protein-ligand complexes. The virtual screening revealed that two alkaloidal metabolites, camptothecin and GKK1032A2 showed excellent binding energy with any of these target proteins compared to reference commercial fungicides, azoxystrobin and strobilurin. Of the detected compounds, GKK1032A2 bound to both target proteins of *M. oryzae*. Both compounds showed excellent bioactivity scores as compared to the reference fungicides. Results of our computational biological study suggest that both camptothecin and GKK1032A2 are potential fungicides that could also be considered as lead compounds to design novel fungicides against the blast fungus. Furthermore, the GKK1032A2 acted as a multi-site mode of action fungicide against *M. oryzae*.

## 1 Introduction

Blast disease caused by the filamentous fungus, *Magnaporthe oryzae* (anamorph: *Pyricularia oryzae*), is one of the most destructive diseases of cereal crops including rice, wheat, barley, finger millet and maize [11,62]. The disease is a potential threat to wheat cultivation worldwide. This fearsome disease causes a major loss of about 10–30% of the world's rice production [15]. Rice blast is notorious amongst the rice dependent regions of the world. The same cannot be said for wheat blast, as it used to be confined only in South American countries such as Brazil, Bolivia, Argentina and Paraguay until 2015. The disease was first time reported in an Asian country, Bangladesh, in February 2016 [21,22]. Districts in India that are near to the wheat blast contaminated regions of Bangladesh have also been reported by the news to contain the disease. Even in the far reaches of Africa, the country Zambia, was reported to have been contaminated by the wheat blast disease [54]. The method of the disease spread is either air-borne if short distance, or the trading of contaminated seeds/grain for long distance spread [36,51]. Although the rate of devastation by the blast disease depends on several factors, however, yield loss can be raised up to 100% in a favorable environment to the fungus [11,14].

The species of *M. oryzae* consists of several host-specific pathotypes [18]. The pathotypes are classified as the *Oryzae* pathotype for infecting rice, the *Triticum* pathotype for infecting wheat, the *Setaria* pathotype for infecting foxtail millet, the *Lolium* pathotype for infecting ryegrass and other variants [23,56]. Even though the isolates are genetically distinct from another due to coming from different hosts, cross-infection is still a possibility [10,51]. The isolate of wheat from *M. oryzae* is able to infect barley, maize, triticale, durum wheat and swamp rice grass in laboratory conditions, while the *M. oryzae* isolates of rice can cause disease in wheat under certain conditions [21,48,49]. The virulence of the pathogen for cross-infection in field conditions have yet to be confirmed.

The control of blast disease is difficult and mainly achieved through the use of chemical fungicides [5,25]. However, extensive use of chemical fungicides has resulted in development of resistance in fungus against the fungicides [5,11,45]. Resistance sources for breeding blast resistance are limited in some cases such as in wheat. Due to the rapid speed in evolution of the pathogen, traditional breeding strategies take more time to develop resistance [18,52]. Therefore, development of new fungicides using bioactive natural compounds with novel mode of actions is a promising approach to managing blast disease [24]. Research has been conducted to discover the antifungal natural compounds against blast fungus *in vitro*, but filed efficacy and mode of action of most of these compounds are unclear [6,7,64]. The natural compounds directly affect the fungal cell, while the other compounds act as an inhibitor to the fungal, cellular and metabolic process of the cell's growth. Presently, researchers are focusing on the identification of compounds that have an impact on the fungal cellular process or pathogenicity. The goal is to find the proteins that are responsible for the cellular process or pathogenicity of specific fungi in order to design specific inhibitors to prevent its growth. Like many other fungal pathogens, *M. oryzae* develops in host cells by a specialized protein called single-stranded DNA binding protein and suppresses the host cell immunity by the use of an effector protein. Therefore, both proteins are valuable targets for the development of fungicides. *M*. *oryzae* (Mo) subunit 1 (i.e., *MoSub1*) is an ssDNA binding protein (4AGH) from the rice blast fungus. Sub1/PC4 is an orthologue of this protein [20,63]. A conserved DNA-binding domain in Sub1 and its human orthologue PC4 binds single-stranded DNA or unpaired dsDNA. It binds to single-stranded DNA dT12 tightly with an affinity of 186 nM [20]. In *M. oryzae*, ssDNA binding protein is a multifunctional protein essential for transcription initiation, elongation, and downstream transcription processes [20,63]. When a fungus infects the host cells, effector proteins (6R8M) are released that bind to rice's heavy metal-associated (HMA) domain. These proteins knock out *OsHIPP20*, a susceptibility gene (*S*-gene), and are also responsible for increasing the colonization process and decreasing host immunity [39,44]. By studying the effects of molecular docking and inhibitors on the pathogenic fungus, the potential for inhibitors that prevent such enzymes or proteins from developing can be achieved.

*In silico* analysis of molecular docking and protein-ligand interaction between antifungal metabolites on target enzymes/proteins are crucial to understand their true potential against *M. oryzae*. This study is aimed to display recently reported inhibitory natural products against blast fungus *M. oryzae* to understand their mechanisms of action and promise as candidate fungicides using *in silico* molecular docking studies on some enzymes/proteins involved in infection of plants by the blast fungus. The specific objectives of this study were (i) virtual screening and molecular docking simulation of 39 promising antifungal natural products on ssDNA binding protein and an effector protein using PyRx 0.8; ii) assess fungicide-likeness, and iii) bioactivity of natural compounds using *in silico* analysis.

## 2 Materials and methods

### 2.1 Protein preparation

The crystal structure of *M. oryzae* single-stranded DNA binding protein (PDB ID: 4AGH at 1.79 Å resolution), and effector protein (PDB ID: 6R8M at 1.85 Å resolution) was retrieved from the Research Collaboratory for Structural Bioinformatics (RCSB) Protein Data Bank (PDB) [3] and considered as a template for all molecular docking simulation. For protein preparation, we used Discovery Studio 2019 molecular visualization software 4.5 [50] and PyMOL 2.3.3 software [40]. First, the proteins were uploaded to the software to find some unnecessary objects such as default given ligands, ions, and water molecules. These unnecessary objects were removed from the PDB file. Finally, the files were saved in PDB file format for further analysis.

### 2.2 Ligand dataset preparation

The canonical smiles of 39 compounds viz. 1STD (Cryptocin, Tanzawaic-acid-L, Camptothecin, HDFO), 1YBV (Alternariol-monomethyl-ether, GKK1032A2, Arohynapene-A, Camptothecin, Tricyclazole), 6JBI (Chaetoviridin-A, GKK1032A2, Camptothecin, Rocaglaol), 5E9G (Arohynapene-B, Pannellin) etc. were retrieved from the PubChem database [9,24], and their 3D structures were generated using Online SMILES Translator and Structure File Generator [43]. Afterward, each compound was ready as ligand for molecular docking study with the target proteins. The two dimensional (2D) and three dimensional (3D) chemical structures of best-docked compounds are illustrated in Fig. S1.

### 2.3 Molecular docking simulation

Molecular docking is a well-known and reliable technique in computer-aided drug design (CADD) processes in structural biology [1]. The technique ensures the best prediction of binding mode between a small molecule and a specific macromolecule [41]. The condition for binding to occur spontaneously must be associated with a negative Gibbs' free energy of binding. It is possible to quantify experimentally the strength of an interaction between a protein and ligand based on the free energy of binding through computational method using 3D structures. According to the pharmacology, it is well-known that if an enzymes active site is blocked by ligand, its functional activity will be terminated [24]. The active site of the proteins was identified before going to the molecular docking simulation study through metaPocket (https://projects.biotec.tu-dresden.de/metapocket/) and cross-checked by another server named CASTp (http://sts.bioe.uic.edu/castp/index.html?2r7g). In metaPocket, four methods (LIGSITEcs, PASS, Q-SiteFinder, and SURFNET) work simultaneously to rise the success rate of prediction [19]. Whereas, solvent accessible surface model (Richards' surface) and molecular surface model (Connolly's surface) use in CASTp to locate, delineate and measure geometric and topological properties of protein structure which include area and volume of pocket [46,55].

Molecular docking simulation was carried out using PyRx 0.8 virtual screening software [61]. For simulating the best interaction docking was performed, setting the center in axis x – (−6.4507), axis y – 25.1892, and axis z – 9.0475 with the dimension was in axis x – 30.3597 Å, axis y – 44.1386 Å and axis z – 42.1486 Å for single-stranded DNA binding protein (PDB ID: 4AGH) and center in axis x – (−34.1603), axis y – (−0.7604) and axis z – 7.9994 with the dimension was in axis x – 86.1772 Å, axis y – 61.9035 Å and axis z – 67.7790 Å for effector protein (PDB ID: 6R8M). After docking simulation, the protein data bank partial charge & atom type (pdbqt) file format, given by PyRx as output, was saved for further protein-ligand interaction analysis.

### 2.4 Protein-ligand interaction analysis

For a clear view of protein-ligand interaction of the best-docked complexes, 2D plots of protein-ligand interactions were analyzed through Discovery Studio 4.5. It generates a 2D graph of hydrogen bonds, electrostatic interactions, and hydrophobic interactions, contributing to the affinity of the drug-like molecules within the active site of *M. oryzae* proteins.

### 2.5 Fungicides likeness

The physicochemical parameters of the most promising compounds were predicted using the web tool SwissADME (http://www.swissadme.ch/index.php). The predicted parameters included the number of rotatable bonds, number of hydrogen bond acceptors, number of hydrogen bond donors, partition coefficient log p (miLog P), molecular weight, synthetic accessibility, and topological polar surface area (TPSA).

### 2.6 Bioactivity score prediction

The online Molinspiration Cheminformatics server (http://www.molinspiration.com) was utilized to evaluate the biological activity of selected compounds. The prediction was based on the enzyme inhibition score such as G-protein-coupled receptor (GPCR), Ion channel modulator, Kinase inhibitor, Nuclear receptor ligand, Protease inhibitor, and Enzyme inhibitor. The results are calculated according to previously published recommendations [33]. Therefore, it is recommended that if the value is equal to or greater than 0.00, the more active it will be, while if the values are between −0.50 and 0.00, it is moderately active, and, if the score is less than −0.50, it is be considered inactive [4,29].

### 2.7 Molecular dynamics simulation

To carry out the molecular dynamics simulation of the ligand-protein and the reference compound (Strobilurin)-protein complexes, YASARA (version 22.9.24) and AMBER14 force fields were utilized [26,30]. Prior to optimizing and orienting their hydrogen-bond network, the docked complexes were first cleaned. Utilizing the TIP3P solvation model, a cubic cell of the simulation with periodic boundary conditions was generated [37]. In every direction the simulated cell was expanded by 20 Å from the docked complexes. The physiological conditions were set for the simulation cell which comprised 298 K temperature, 0.9% NaCl (sodium chloride), and pH 7.4. In the simulated annealing scheme, the steepest gradient algorithm (5000 cycles) was applied for the preliminary minimization of energy [38]. A time step of 1.25 fs (fs) was chosen for the simulation technique. With a cutoff radius of 8 Å, the PME (particle-mesh Ewald) scheme was employed for the calculation of the long-range electrostatic interactions [27]. Data from the simulation trajectory were recorded at intervals of every 100 ps (ps). The Berendsen thermostat, together with constant temperature and pressure were used in the simulations for 100 ns (ns). Through the investigation of the simulation trajectory data, the root-mean-square deviation (RMSD), root-mean-square-fluctuation (RMSF), radius of gyration (Rg), hydrogen bond, and solvent-accessible surface area (SASA) were assessed [24,37].

## 3 Results

### 3.1 Molecular docking simulation study

**Fig. 1:**
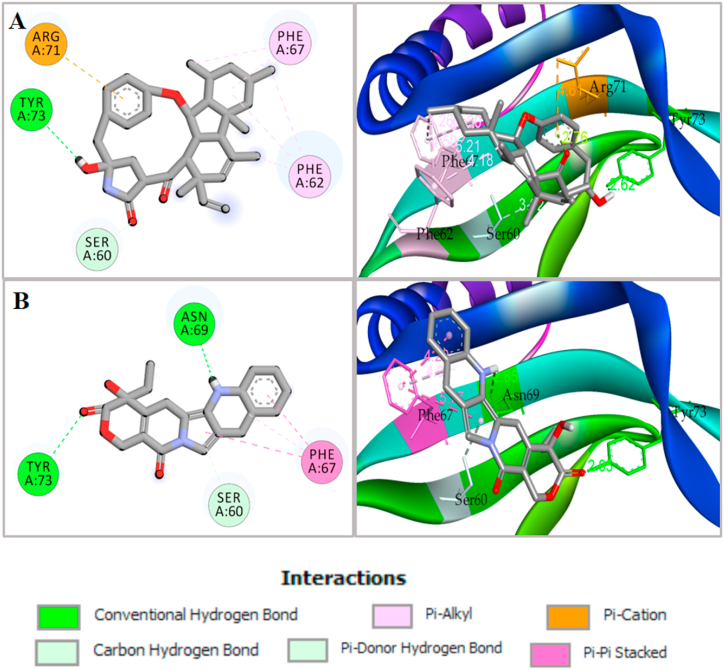
Molecular interactions of the screened compounds. (A) GKK1032A2 docked in complex with single-stranded DNA binding protein (PDB ID: 4AGH). The GKK1032A2 formed a hydrogen bond with A:TYR73 and A:SER60. In addition, A:PHE62 and A:PHE67 formed hydrophobic interactions, and A:ARG71 formed electrostatic interactions. (B) Camptothecin formed a hydrogen bond with A:TYR73, A:ASN69, and A:SER60 and hydrophobic interactions with residues A:PHE67.2D interaction analysis is shown on the left, and 3D interaction analysis is shown on the right side.

Molecular docking simulations were used to clarify the compounds' binding mode and obtain other information that could be utilized for further structural optimization [16,57]. Optimum binding affinity is achieved by lowering binding free energy between the target protein and the lead compound through optimization [13,24,37]. Our selected 39 antifungal natural products and reference fungicide (azoxystrobin and strobilurin) compounds were docked against the two different target proteins, single-stranded DNA binding protein (4AGH) and effector protein (6R8M) (Fig. 1). The docked compounds were ranked based on the maximum occupancy of the binding pocket with minimum free energy, the strength of hydrogen bonding, and other potential non-covalent interactions. Out of 39 docked molecules, top-ranking docked compounds were selected. Protein-ligand binding affinity is essential for biological processes, as these physical and chemical interactions determine biological recognition at the molecular level. In this way, it was possible to look for a ligand capable of inhibiting or activating a specific target protein through its interaction. Therefore, finding a ligand binds to a target protein with high affinity was also ranked [17]. The ranking criteria involved Lipinski rules five, the number of hydrogen bond interactions, and binding with the selected protein targets involving the binding pocket residues. Compounds were docked with the single-stranded DNA binding protein (4AGH). Among the tested compounds, the compound GKK1032A2 (−8.4 kcal/mol) and camptothecin (−8.2 kcal/mol) showed the highest binding affinities to 4AGH (Fig. 1, Table 1A). A hydrogen bond favors the docking interaction of GKK1032A2 with A:TYR73 and A:SER60, while non-bonded hydrophobic interaction with A:PHE62 and A:PHE67 and electrostatic interaction with A:ARG71 (Fig. 1A, Table 1A). The compound camptothecin showed hydrogen bond interaction with A:TYR73, A:ASN69, and A:SER60, while hydrophobic interaction with A:PHE67 (Fig. 1B, Table 1A). Both compound GKK1032A2 and camptothecin showed better interaction with target protein than reference compound Azoxystrobin and Strobilurin [24].

**Table 1:** Summary of the top-ranked compounds screened against single-stranded DNA binding protein (4AGH) and effector protein (6R8M) with their respective binding energy and interacting amino acid residues.

Compounds	Binding Energy (kcal/mol)	Residues involved in Hydrogen bond Interaction	Residues involved in Hydrophobic interaction	Electrostatic interaction
A. 4AGH				
GKK1032A2	−8.4	**A:TYR73, A:SER60**	A:PHE62, A:PHE67	**A:ARG71**
Camptothecin	−8.2	**A:TYR73, A:ASN69, A:SER60**	A:PHE67	
Reference (Azoxystrobin)	−6.9	**A:SER60, A:ASN69, A:ARG71**	A:PHE62, **A:PRO82**	
Reference (Strobilurin)	−7.3	A:GLN93	**A:PRO82**, A:PHE67	
B. 6R8M				
GKK1032A2	−8.2	F:LYS194, **G:LYS79**	F:LYS194	
Reference (Azoxystrobin)	−6.4	F:ARG246, F:LEU254, G:LYS79	G:TRP84, F:LEU254, F:VAL257, F:ILE242,	
Reference (Strobilurin)	−7.2	**C:GLY102, C:TRP103, C:PHE99**	**C:PRO101**, F:ALA244, F:LEU241, F:LYS240	

Bold form indicates the active site amino acid.

**Fig. 2:**
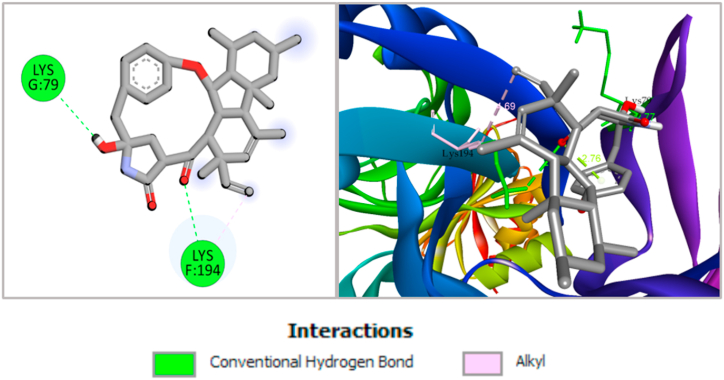
Molecular interactions of the docked compounds. GKK1032A2 docked in complex with effector protein (PDB ID: 6R8M). It shows hydrogen bond with residues F:LYS194 and G:LYS79 hydrophobic non bonded interactions were formed with F:LYS194. The 2D interaction analysis is shown on the left, and 3D interaction analysis is shown on the right side.

In the case of the effector protein (6R8M), compound GKK1032A2 had the highest binding affinity (−8.2 kcal/mol) amongst all compounds (Fig. 2, Table 1B). The rest of the compounds had binding affinity > -8.0 kcal/mol. The docking interaction showed a hydrogen bond with F:LYS194 and G:LYS79 (Fig. 2), whereas hydrophobic interaction with F:LYS194 is listed in Table 1B. Bond distance and type of interactions of the docked compounds are shown in Table 2. Our selected compounds possessed highest binding energy compare to reference compound Azoxystrobin and Strobilurin.

**Table 2:** Type of interactions, interacting residues, and bond distance of single-stranded DNA binding protein (4AGH) and effector protein (6R8M) with the two best binding energy compounds.

Compounds	Interacting amino acid residues	Bond distance (Å)	Interaction category	Type of Interaction
4AGH vs. GKK1032A2	A:SER60	3.01869	H Bond	Carbon H Bond
	A:PHE62	5.20831	Hy Bond	Pi-Alkyl
	A:PHE67	4.19531	Hy Bond	Pi-Alkyl
	A:TYR73	2.6156	H Bond	Conventional H Bond
4AGH vs. camptothecin	A:TYR73	2.95224	H Bond	Conventional H Bond
	A:ASN69	2.85068	HBond	Conventional H Bond
	A:SER60	3.34247	HBond	Pi-Donor H Bond
	A:PHE67	4.19669	Hy Bond	Pi-Pi Stacked
6R8M vs. GKK1032A2	G:LYS79	2.76527	H Bond	Conventional H Bond
	F:LYS194	2.83671	H Bond	Conventional H Bond
	F:LYS194	4.6876	Hy Bond	Alkyl

H = Hydrogen, Hy = Hydrophobic.

### 3.2 Fungicide likeness

Physicochemical properties of the potential compounds were analyzed to evaluate their fungicide-likeness nature. We analyzed the fungicide-likeness of the natural compounds under the well-established fundamental rule of drug-likeness, i.e., Lipinski rule of 5. Natural compounds' physicochemical properties include molecular weight, number of rotatable bonds, number of hydrogen bond acceptors, number of hydrogen bond donors, topological polar surface area, Fraction Csp3, Molar refractivity, and Synthetic accessibility were analyzed. The predicted results are shown in Table 3. Interestingly, the selected natural compounds bear the molecular weight ranged from 503.67 g/mol to 348.35 g/mol. The miLogP values of the potential compounds were 5.08 and 2.03. According to Lipinski's rule, most “drug-like” molecules have many hydrogen bond acceptors ≤10 and many hydrogen bond donors ≤5. Furthermore, the number of hydrogen bond donors was less than five, and the number of hydrogen bond acceptors was less than 10. Besides, TPSA (topological polar surface area) of the potential compounds was observed in the 75.63 Å^2^ and 81.2 Å^2^.

**Table 3:** Physicochemical properties of selected potential compound and reference fungicide compound.

Compound	MW (g/mol)	RB	HBA	HBD	miLogP	Lipinski	TPSA (Å^2^)	Synthetic accessibility
GKK1032A2	503.67	1	2	4	5.08	Yes	75.63	7.28
Camptothecin	348.35	1	5	1	2.03	Yes	81.2	3.84
Azoxystrobin	403.39	8	8	0	3.38	Yes	103.56	3.42
Strobilurin	442.5	6	7	1	4.92	Yes	83.45	5.6

MW- Molecular weight, RB- Rotatable Bond, HBA- Hydrogen bond acceptor, HBD- Hydrogen bond donor, TPSA- Topological surface area.

### 3.3 Bioactivity score assessment of selected potential natural products

The bioactivity scores of the selected natural compounds were predicted through the Molinspiration server. In this prediction, biological activity of the compounds measured by the bioactivity score as an inhibitor of the targeted proteins (Table 4), which were classified into three different ranges: molecule having a bioactivity score greater than 0.00 was most likely to illustrate meaningful biological activity, while scores extending from −0.50 to 0.00 were expected to be moderately active, and if the score was less than −0.50, it was presumed to be inactive. The bioactivity scores for the G protein-coupled receptor ligand (GPCR) were most active for camptothecin, and moderately active for GKK1032A2. On the other hand, the ion channel modulators' scores for both compounds were moderately active. The result of kinase inhibitors scores for the compound camptothecin had biological active score values, but the compound GKK1032A2 was inactive. Moreover, the nuclear receptor score values, both compounds were biologically active according to the classification ranges of Linn et al. [33]. For protease inhibitors, compound GKK1032A2 had biologically active score values, whereas compound camptothecin had moderately active scores. The structures of both compounds had score values for enzyme inhibitors greater than 0.00, considered biologically active.

**Table 4:** Prediction of bioactivity of the selected compounds and reference fungicide compounds.

Compounds	GPCR ligand	Ion channel modulator	Kinase inhibitor	Nuclear receptor ligand	Protease inhibitor	Enzyme inhibitor
GKK1032A2	−0.16	−0.17	−0.76	0.13	0.07	0.05
Camptothecin	0.46	−0.15	0.27	0.07	−0.1	1.11
Azoxystrobin	0.25	0.03	0.09	0.25	−0.12	0.19
Strobilurin	0.27	0	−0.17	0.51	−0.03	0.17

### 3.4 Molecular dynamics simulation

**Fig. 3:**
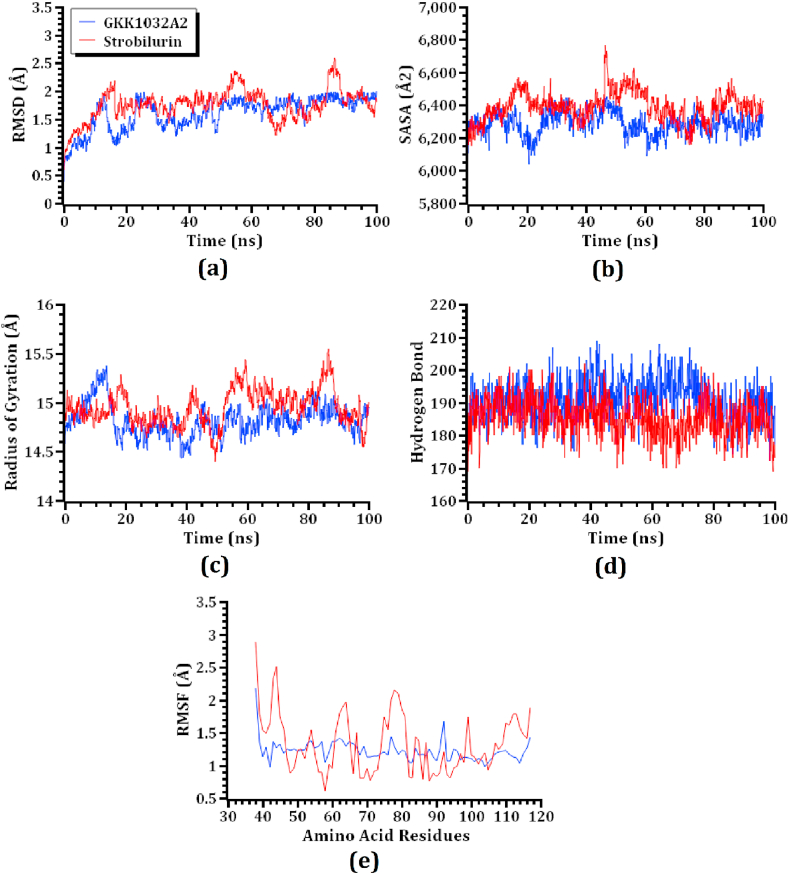
Molecular dynamics simulations of the GKK1032A2-4AGH and Strobilurin-4AGH complex indicated, in alphabetical order, (a) root mean square deviation, (b) solvent accessible surface area, (c) radius of gyration, (d) number of hydrogen bonds, and (e) root mean square fluctuation.

**Fig. 4:**
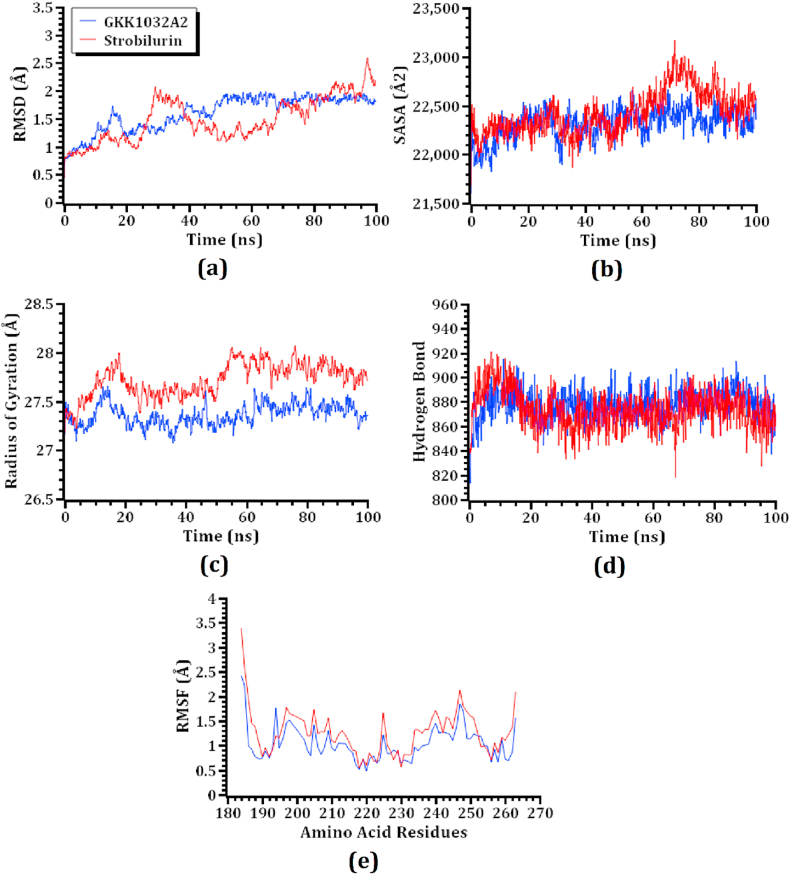
Molecular dynamics simulations of the GKK1032A2-6R8M and Strobilurin-6R8M complex indicated, in alphabetical order, (a) root mean square deviation, (b) solvent accessible surface area, (c) radius of gyration, (d) number of hydrogen bonds, and (e) root mean square fluctuation.

For exploring the structural inflexibility and validating the docking scenarios of the topmost ligand-protein complexes GKK1032A2-4AGH and GKK1032A2-6R8M as well as the reference compound (Strobilurin) with both the proteins, molecular dynamics simulations were performed for 100 ns. By analyzing the C-alpha atom's RMSD, the firmness of the ligand-protein complexes was assessed. In the case of both the 4AGH and 6R8M protein, the GKK1032A2-4AGH, Strobilurin-4AGH, GKK1032A2-6R8M, and Strobilurin-6R8M complex all displayed an early rise in the RMSD value (Fig. 3, Fig. 4a). The Strobilurin-4AGH complex was unstable over the simulation period, as evidenced by the fact that the RMSD of the Strobilurin-4AGH complex was, on average, greater than the GKK1032A2-4AGH complex and it also exhibited major fluctuations during the simulation period compared to the GKK1032A2-4AGH complex that stabilized around 50 ns and remained stable for the rest of the simulation period with minor fluctuations and indicated its stability (Fig. 3a). Similarly, the RMSD of the Strobilurin-6R8M complex displayed significant fluctuations during the simulation time compared to the GKK1032A2-6R8M complex that stabilized around 50 ns and persisted stably with negligible fluctuations to the rest of the simulation time and designated the firmness of the GKK1032A2-6R8M complex during the simulation time (Fig. 4 a).

The SASA (solvent accessible surface areas) data were retrieved to inspect how the surface areas of the 4AGH and 6R8M proteins changed in response to the interaction with the GKK1032A2 and Strobilurin compounds. SASA is an important measure for accessing protein stability and folding since greater SASA values suggest an enlarged protein surface area, whereas lower SASA values reflect a decreased protein surface area (Savojardo et al., 2011). From 5 ns to almost 100 ns of the simulation period, the SASA of the Strobilurin-4AGH complex was higher than the GKK1032A2-4AGH complex, suggesting that the Strobilurin-4AGH complex had a larger surface area during the simulation (Fig. 3b). Likewise, the Strobilurin-6R8M complex's SASA value was greater between 55 and 100 ns of the simulation time than the GKK1032A2-6R8M complex, signifying the enlarged surface area of the 6R8M protein in response to the interaction with the Strobilurin compound (Fig. 4b).

For the evaluation of the stiffness or lability of the ligand-protein complexes, the Rg values were assessed where stiffer ligand-protein complexes were determined by lower Rg values and more labile ligand-protein complexes were recognized by higher Rg values [38]. The Strobilurin-4AGH complex displayed an initial decrease in the Rg value, whereas the GKK1032A2-4AGH complex showed an initial increase in the Rg values (Fig. 3c). In the case of the 6R8M protein, both the GKK1032A2-6R8M and Strobilurin-6R8M complex exhibited a preliminary decrease in the Rg values (Fig. 4c). The Rg value of the GKK1032A2-4AGH complex was lower on average than the Strobilurin-4AGH complex which signified the rigidness of the GKK1032A2-4AGH complex during the simulation period. Likewise, the Rg of the Strobilurin-6R8M complex was significantly higher approximately throughout the whole simulation time, indicating the lability of the Strobilurin-6R8M complex than the GKK1032A2-6R8M complex.

Hydrogen bonds play a crucial for maintaining the firmness and integrity of the docked ligand-protein complexes throughout the simulation period and so the complexes' hydrogen bonds were assessed. The GKK1032A2-4AGH, Strobilurin-4AGH, GKK1032A2-6R8M, and Strobilurin-6R8M complex all formed a greater number of hydrogen bonds during the simulation but the GKK1032A2-4AGH and GKK1032A2-6R8M complex exhibited better hydrogen bonds on average than the Strobilurin-4AGH and Strobilurin-6R8M complex respectively which designated the integrity of these complexes throughout the simulation (Fig. 3, Fig. 4d). The flexibility of the 4AGH and 6R8M protein across the amino-acid residues throughout the simulation time were scrutinized by evaluating the RMSF value. An increase in rigidity is generally accompanied by a decrease in RMSFs [38]. The RMSF value for the GKK1032A2-4AGH and Strobilurin-4AGH complex was below 2.5 Å and 3 Å respectively (Fig. 3e). The RMSF value for the GKK1032A2-4AGH was less fluctuating compared to the Strobilurin-4AGH complex which suggested their higher inflexibility. The GKK1032A2-6R8M complex's RMSF value fluctuated not as much as the Strobilurin-6R8M complex, indicating more rigidity of the GKK1032A2-6R8M complex compared to the Strobilurin-6R8M complex (Fig. 4e).

## 4 Discussion

The blast disease caused by *M. oryzae* is a destructive plant disease of major cereal crops including rice, wheat and maize that causes enormous economic losses by reducing grain yield worldwide [52]. Although the chemical fungicides provide marginal protection against blast disease, increased risk of health and environmental hazards and development of resistance are a matter of concern. Discovery of natural products with novel mode of actions is warranted for the control of this notorious fungal phytopathogen [24]. Recently, Chakraborty et al. comprehensively reviewed the bioactive natural products that significantly inhibit various pathotypes of *M. oryzae* both *in vitro* and *in* vivo [7]. It appears from that review that almost all of those natural products have not been elaborately studied for their suitability as fungicides or lead compounds against the blast fungus. To find out potential fungicide candidates against *M. oryzae*, we selected the potential 39 compounds that inhibit the growth of *M. oryzae* fungus differently for an *in-silico* study to assess their molecular docking and bioactivity scores against two proteins viz ssDNA binding protein (4AGH) and AVR-Pik effector (6R8M) in the fungus essential for the disease development in host plants. Our molecular docking study revealed that at least two natural compounds viz. camptothecin and GKK1032A2 strongly bind either 4AGH or 6R8M proteins responsible for transcription in fungus or suppression of the host immunity. As these two proteins are critical for infection of plants by *M. oryzae* fungus, the two identified natural products might be useful as potential fungicide candidates or lead compounds to develop novel fungicides against the blast fungus.

*M. oryzae* ssDNA binding protein (4AGH) Mosub1 is an orthologue of yeast ssDNA binding protein Sub1 or human PC4. Its most likely function relies on the fungus development [20]. Though much research is needed to understand the proper functions of Mosub1, the functions of its orthologues (sub1/pc4) are well documented [20,63]. Subunit Sub1/PC4 has many functions in DNA metabolic activities, such as transcription and DNA repair, and phosphorylation substantially impacts their DNA binding capacity. In particular, they are involved in transcription initiation, elongation, splicing, termination, and genome stability maintenance and have positive and negative roles [63]. These enzymes can be promising molecular targets for identifying potential inhibitors because they are involved in developing fungus that creates disease in plants. AVR-Pik is an effector protein (6R8M) found in the rice blast fungus *M. oryzae* and has also been shown to be a good target for designing fungicides. Effector proteins are secreted in the host cells upon infection by the fungus. The effector protein functions as a suppressor of immunity and speeds up the colonization process by overcoming the host defense systems [39]. AVR-Pik binds to rice's heavy metal-associated (HMA) domain and knocks out OsHIPP20, a susceptibility gene (S-gene). Which, in this case, serves to suppress immunity [44]. However, a trade-off also occurs when plant nucleotide-binding, leucine-rich repeat (NLR) receptors (Pik1 and Pik2) recognize and detect AVR-Pik effector protein and activate an immune response [12,39]. In the present study, 39 compounds were subjected to a molecular docking study, and only 2 (GKK1032A2 and camptothecin) of them showed a good binding affinity with the two proteins mentioned above of *M. oryzae*.

One of the interesting findings of this study is that both GKK1032A2 and camptothecin strongly bind with ssDNA binding protein (4AGH), whereas GKK1032A2, showed a solid binding affinity for effector protein (6R8M) with less energy requirement compared to commercial fungicides, strobilurins and azoxystrobin. Lipinski's rule of 5 was then employed to analyze the fungicide-likeness of these tow natural compounds. The molecular weight of the selected compounds was more or less than 500 g/mol (348.35 g/mol and 503.67 g/mol). A chemical's molecular weight is an essential criterion in determining its fungicides activity. The molecules with low molecular weight (<500) are readily transported, diffused, and absorbed by the cell membrane compared to large molecules [32]. In addition, the positive Log*P* values indicate an easier passage of compounds through bio-membranes, and the acceptable limit is <5 [8,47]. Taken together, these results suggest that both camptothecin and GKK1032A2 are promising fungicide candidates that merit further in vivo evaluation for blast control either in the green house or in the field.

The lipophilic compounds are easily permeable through the cell membrane by passive diffusion and bind with biomolecules to inhibit the vital metabolic enzymes in the cell. Therefore, the membrane permeability depends on the lipophilic nature of a compound. Importantly, the calculated log P values of the selected natural compounds were 2.03 and 5.08 that are ideal for crossing the cell membrane of the fungus. Recently, Steinberg et al. [53] reported that C_18_–SMe_2_^+^, a mono-alkyl lipophilic cation (MALCs) having a Log*P* value of 2.26, readily diffuses through the plasma membrane. In this study, the molecular weight and logP value of one compound surpassed the expected limit mentioned in Lipinski s rule of 5, and this slightly increased molecular weight and miLogP value may not significantly influence the transportation and diffusion of this natural compound. It has been shown that the molecular mass of several FDA-approved drugs was more significant than 500 g/mol [42]. Furthermore, the number of hydrogen bond donors was less than five, and the number of hydrogen bond acceptors was less than 10 [58]. Besides, TPSA of the potential compounds was observed at 75.63 and 81.20 Å^2^ which is also between the acceptable ranges [34]. In the bioactivity scores, the two selected compounds, camptothecin and GKK1032A2 poses a score value of - 0.50 to 0.00, which indicates the compounds are biologically active.

Among the selected antifungal compounds, the plant originated alkaloid camptothecin showed a strong binding affinity with 4AGH. A series of earlier studies reported that camptothecin treatment can inhibit the growth of *M. oryzae*, *Rhizoctonia solani*, *Alternaria alternata*, *Colletotrichum gloeosporioides*, *Fusarium oxysporum*, *Botrytis cinerea*, *Sphaerotheca fuliginea*, and *Pseudoperonospora cubensis* [28,59,62]. In this study, camptothecin was as the most effective natural compounds against *M. oryzae* with a lower concentration of 1.53 μg/mL (EC50 value). In addition, this compound was effective against the mycelial growth of *A. alternate* and *F. oxysporum* with an EC50 value of 250 μg/mL, and for *C. gloeosporioides*, it was 500 μg/mL. The molecular simulation result showed that CPT could bind to the interface of the DNA-topoisomerase I complex of *M. oryzae* and affect the translation and carbohydrate metabolism/energy metabolism leading to cell death [59]. The other natural compound selected in this study, GKK1032A2, was discovered from the fungus *Penicillium* sp. which showed conidial germination inhibition in *M. oryzae* [44]. Compound GKK1032A2 was effective in inhibiting *M. oryzae* at a concentration of 3 μg/mL. However, it is ineffective against other phytopathogenic fungi, including *F. graminearum*, *B. cinerea*, and *P. infestans* [2]. Therefore, the selected two natural compounds by this study could be used as lead compounds or biofungicide to inhibit the aforementioned proteins in *M. oryzae*.

Despite a plethora of fungicides are available to control many diseases, the blast disease caused by the different pathotypes of *M. oryzae* remains to be managed effectively. A series of recent studies suggest that *M. oryzae* has already acquired resistance against numerous chemical fungicides [5,31,35]. The commercially available fungicides that are currently being used generally target a single enzyme which can be overcome by single point mutation [5,60]. For instance, extensive use of strobilurin (QoI) fungicides in Brazil has led to the widespread distribution of cyt *b* mutations conferring resistance in strains isolated from wheat and other grasses [5]. Therefore, fungicides having multi-site mode of action are needed to be discovered to manage devastative blast disease [31]. The present study identified two potential biofungicides, among which GKK1032A2 had inhibitory capacity to modulate both the proteins evaluated. Therefore, this compound merit further *in vivo* evaluation for considering it as a potential fungicide or lead compound to control blast disease.

## 5 Conclusion

The control of blast disease using natural compounds is an advanced and risk-free method for disease management. The results of the present study revealed that camptothecin and GKK1032A2 are potential fungicides or lead compounds to control blast fungus *M. oryzae*. However, the compound GKK1032A2 showed the most significant binding affinity for both target proteins and it had many H-bonds and bioactivity scores. Therefore, this compound showed the best results than the other 39 compounds tested in this computational investigation. Furthermore, compound GKK1032A2 could cross cell membranes and be able to inhibit the target proteins in *M. oryzae*, which are involved in pathogenesis-related factors. Taken together, our results indicate that the natural antifungal compound, GKK1032A2 targets both proteins involved in ssDNA binding protein and the effector protein of the blast fungus *M. oryzae*. To the best of our knowledge, this computational study for the first time elaborated the antifungal mechanism of actions of two natural compounds, camptothecin and GKK1032A2 against the cereal blast fungus *M. oryzae*. Further *in vivo* molecular and field studies are required to confirm these results of the *in silico* study before recommending camptothecin and GKK1032A2 as fungicides against *M. oryzae*.

## Author contribution statement

Md. Abdullah Al Mamun Khan; Asif Ahsan; Md. Arif Arif Khan; Jannatul Maowa Sanjana; Suvro Biswas; Md. Abu Saleh; Tahsin Islam Sakif: Performed the experiments; Analyzed and interpreted the data; Contributed analytical tools or data; Wrote and revised the manuscript.

Dipali Rani Gupta; M. Nazmul Hoque; Md. Masuder Rahman: Analyzed and interpreted the data; Wrote and edited the manuscript.

Tofazzal Islam: Conceived and designed the experiments; Analyzed and interpreted the data; Contributed materials, analytical tools or data; Wrote, edited and critically reviewed the manuscript.

## Funding statement

This research did not receive any specific grant from funding agencies in the public, commercial, or not-for-profit sectors.

## Data availability statement

Data included in article/supplementary material/referenced in article.

## Declaration of interest's statement

The authors declare that they have no known competing financial interests or personal relationships that could have appeared to influence the work reported in this paper.
