# Supplementary material for: In-silico prediction of highly promising natural fungicides against the destructive blast fungus Magnaportheoryzae
Source: Heliyon. 2023 Apr 10;9(4):e15113. doi: 10.1016/j.heliyon.2023.e15113 (PMC10130775; doi:10.1016/j.heliyon.2023.e15113)
Supplement: Multimedia component 1 [file mmc1.docx]

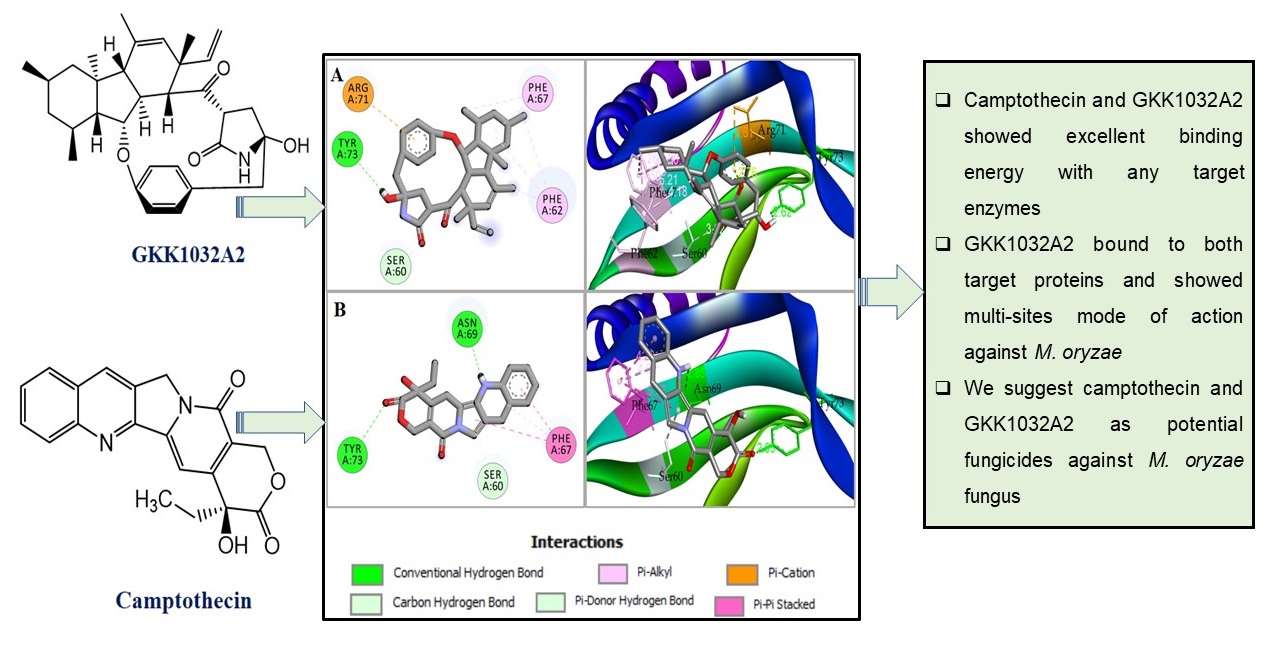


**Figure S1:** Graphical abstract showing the overview of the study. Thirty-nine natural compounds inhibiting the growth of *M. oryzae* were used for docking analysis, and of these compounds, Camptothecin and GKK1032A2 showed excellent binding energy with any target proteins suggesting them as potential fungicides against the worrisome *M. oryzae* fungus.
