# Supplementary material for: In-silico prediction of highly promising natural fungicides against the destructive blast fungus Magnaportheoryzae
Source: Heliyon. 2023 Apr 10;9(4):e15113. doi: 10.1016/j.heliyon.2023.e15113 (PMC10130775; doi:10.1016/j.heliyon.2023.e15113)
Supplement: Multimedia component 2 [file mmc2.docx]

**Supplementary Tables**

**Table S1:** Type of interactions, interacting residues and bond distance of single stranded DNA binding protein (4AGH) and effector protein (6R8M) with the selected fungicide compound.

| **Compounds** | **Interacting amino acid residues** | **Bond distance (Å)** | **Interaction category** | **Type of Interaction** |
| --- | --- | --- | --- | --- |
| 4AGH vs. azoxystrobin | A:SER60 | 2.7307 | H Bond | Conventional H Bond |
|  | A:PHE62 | 3.96388 | Hy Bond | Pi-Pi Stacked |
|  | A:ASN69 | 3.16412 | H Bond | Conventional H Bond |
|  | A:ARG71 | 3.12246 | H Bond | Conventional H Bond |
|  | A:PRO82 | 4.47426 | Hy Bond | Alkyl |
| 4AGH vs. Strobilurin | A:PHE67 | 4.46807 | Hy Bond | Pi-Alkyl |
|  | A:PRO82 | 4.81113 | Hy Bond | Alkyl |
|  | A:GLN93 | 2.6497 | H Bond | Conventional H Bond |
| 6R8M vs. Azoxystrobin | G:LYS79 | 2.83173 | H Bond | Conventional H Bond |
|  | G:TRP84 | 4.41521 | Hy Bond | Pi-Pi Stacked |
|  | F:ILE242 | 4.7518 | Hy Bond | Pi-Alkyl |
|  | F:ARG246 | 2.33609 | H Bond | Conventional H Bond |
|  | F:LEU254 | 2.61158 | H Bond | Conventional H Bond |
|  | F:LEU254 | 5.29513 | Hy Bond | Pi-Alkyl |
|  | F:VAL257 | 5.0804 | Hy Bond | Pi-Alkyl |
| 6R8M vs. Strobilurin | C:PHE99 | 2.63527 | H Bond | Conventional H Bond |
|  | C:PRO101 | 3.93373 | Hy Bond | Alkyl |
|  | C:GLY102 | 2.12499 | H Bond | Conventional H Bond |
|  | C:TRP103 | 2.79058 | H Bond | Conventional H Bond |
|  | F:LYS240 | 3.93216 | Hy Bond | Alkyl |
|  | F:LEU241 | 4.49805 | Hy Bond | Alkyl |
|  | F:ALA244 | 3.47837 | Hy Bond | Alkyl |

H= Hydrogen, Hy= Hydrophobic
